# Arteriovenous Fistula: An Unusual Complication of Orthognathic Surgery—Case Report and a Brief Review

**DOI:** 10.1155/crid/7742546

**Published:** 2025-05-02

**Authors:** Josue Gallardo-Caudillo, Freddy Andrés Vivero-Alcivar, Cinthya María Quisiguiña-Salem, Sergio Esquivel-Martin

**Affiliations:** ^1^Department of Oral and Maxillofacial Surgery, Centro Medico Naval, Mexico City, Mexico; ^2^Department of Oral and Maxillofacial Surgery, Postgraduate and Research Division, Dental School, National Autonomous University of Mexico, Mexico City, Mexico; ^3^Oral and Maxillofacial Surgeon, San Pedro Garza, Nuevo Leon, Mexico

**Keywords:** arteriovenous fistula, dentofacial deformities, lefort osteotomy, orthognathic surgery, postoperative complications

## Abstract

Arteriovenous fistulas rarely occur in the head and neck region. They typically arise from blunt or penetrating trauma but can develop as an uncommon complication following bimaxillary orthognathic surgery, a procedure to correct jaw deformities. This report describes a male patient who experienced an arteriovenous fistula after orthognathic surgery. We detail the diagnosis through patient symptoms and imaging, along with successful treatment via endovascular embolization. We also follow the patient for 1 year. This case highlights the importance of recognizing this rare complication to ensure prompt diagnosis and intervention. We discuss key points for preventing, diagnosing, and effectively treating arteriovenous fistulas after orthognathic surgery.

## 1. Introduction

Le Fort I osteotomy is one of the most common procedures performed by maxillofacial surgeons because of its versatility in correcting deformities and the predictability of its results [1]. Common complications arising after this surgery include bleeding, alterations of maxillary nerve branches, dental injury, maxillary sinusitis, and malocclusion. Rarer and more significant complications include avascular necrosis of the jaw (0.2%), unfavorable fractures or skull base fractures (0.197%), ophthalmic complications (0.019%), and vascular complications such as arteriovenous fistulas (AVFs) and pseudoaneurysms, whose overall prevalence has not been established due to the limited number of reported cases [1, 2].

Most AVFs are caused by facial trauma [3–5] and, in rare cases, can occur after surgical procedures in the maxillofacial area, especially Le Fort I osteotomy [6]. Only 7.8% of maxillofacial AVFs occur after orthognathic surgery, with 5% specifically involving the internal maxillary artery [4].

This case report describes a rare AVF complication following orthognathic surgery in a male patient, successfully treated with endovascular embolization, emphasizing the importance of recognizing and promptly addressing this condition.

## 2. Case Report

**Figure 1:**
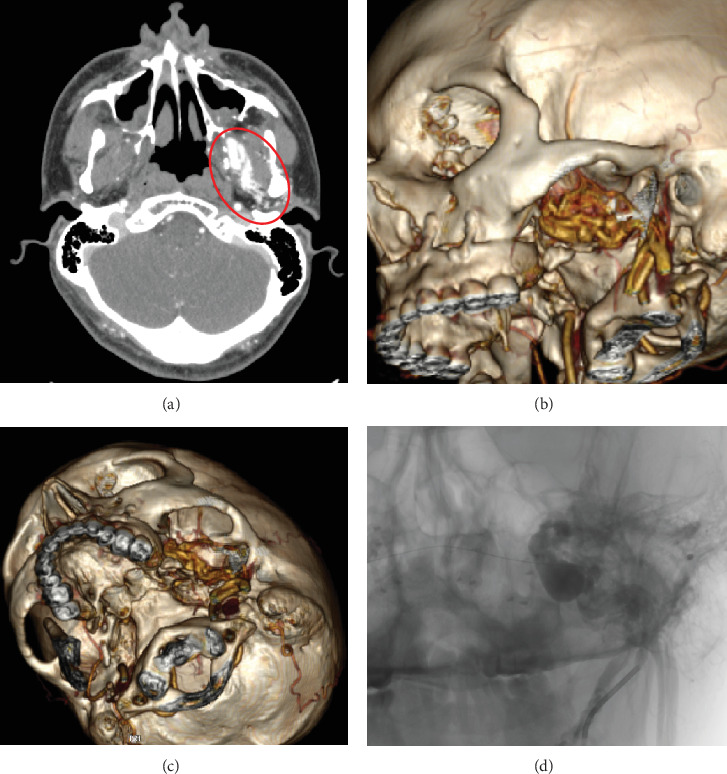
(a) Axial view of the contrast-enhanced CT with hyperdense zone at the pterygomaxillary space/red circle). (b, c) 3D reconstruction with colored blood vessels showing the size of the arteriovenous fistula in lateral view and bottom view, respectively. (d) Angiogram with arteriovenous fistula between right maxillary artery and pterygoid plexus.

We present the case of a 30-year-old male patient with no relevant medical history and no previous surgery in the head and neck region, who was diagnosed with a Class II dentofacial deformity. He underwent bimaxillary orthognathic surgery, using a conventional approach and the maxillary first sequence, with LeFort I osteotomy followed by bilateral sagittal split osteotomy (BSSO). During the pterygomaxillary disjunction on the left side, profuse arterial bleeding occurred and was immediately controlled with local pressure. Postoperatively, the patient reported a palpable thrill/fremitus and a pulsating sensation on the left side. Over the next 5 months, he continuously perceived the thrill/fremitus, which was more noticeable at night and increased in intensity and frequency with his heart rate and physical exertion. The thrill/fremitus was also audible upon auscultation. Consequently, a contrast-enhanced CT scan of the facial bones was performed, revealing a contrast-enhancing mass in the left internal maxillary artery (Figure 1).

The patient was referred to interventional radiology and vascular surgery for fistula embolization. The procedure was performed in a hybrid hemodynamics room under sedation and local anesthesia administered to the right inguinal region, using fluoroscopic guidance.

**Figure 2:**
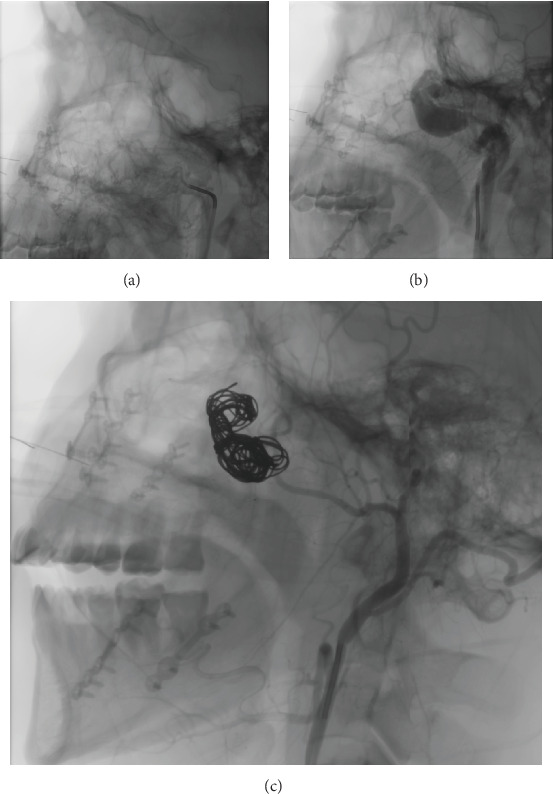
(a) Catheter in the second segment of the left maxillary artery where the fistula was located. (b) Contrast advancing through the arteriovenous fistula. (c) Control angiography with a deposit of contrast medium to confirm that the fistula has been completely obstructed by coils.

A 5Fr introducer was placed via femoral puncture, and the external carotid artery was cannulated using a hydrophilic guidewire, advanced to the internal maxillary artery (Figure 2a). Selective angiography revealed a high-flow AVF in the second segment of the internal maxillary artery, communicating with the pterygoid venous plexus (Figure 2b). Once the fistula-containing segment of the internal maxillary artery was identified, multiple coils were deployed to occlude the feeding artery. Successful occlusion was confirmed by control angiography (Figure 2c). Contralateral angiography was also performed to exclude any connections on the opposite side, none of which were identified.

Upon recovery from anesthesia, the patient reported immediate symptom relief. At the 1-year follow-up, the patient remained asymptomatic and without complications.

## 3. Discussion

Le Fort I osteotomy is a highly versatile and predictable procedure, making it one of the most common surgeries performed by maxillofacial surgeons for correcting deformities [1, 6].

Like any surgery, maxillary osteotomy can lead to a range of postoperative complications, from common issues such as bleeding and infection to rarer occurrences [2, 6]. The pterygomaxillary fossa is a critical area during Le Fort I osteotomy, particularly during the downfracture maneuver, due to the presence of vessels vulnerable to injury, such as the ascending and descending palatine arteries. The pterygoid plexus, a venous network that drains into the cavernous sinus, inferior ophthalmic vein, and facial vein, may also have venous connections with the nasal venous plexus [5, 6].

Multiple cadaveric and tomographic studies have investigated safe distances for pterygomaxillary disjunction using chisels, estimating a safe range of 8.9–17.07 mm. The literature also suggests using a 10-mm curved chisel with a transoral approach for this disjunction. A transverse technique has also been described, where the chisel placement angle relative to the midpalatal plane varies between 64° and 84°, depending on the presence of the third molar, to help reduce the risk of injury to structures within the pterygomaxillary fossa [7].

AVFs are an uncommon complication following surgical procedures, more frequently associated with facial trauma caused by blunt or penetrating objects [3–5, 8]. Goffinet et al. reported that only 7.8% of maxillofacial AVFs occur after orthognathic surgery, with 5% specifically involving the internal maxillary artery [4]. In this case, the AVF was related to the second segment of the maxillary artery. To date, no studies have evaluated the relationship between maxillary artery segments and traumatic vascular lesions, likely due to the limited number of reported cases. Traumatic AVFs are caused by incomplete artery laceration with concomitant laceration of a vein. Incomplete arterial wall rupture results in hemorrhage and hematoma, which eventually blocks the bleeding [5]. It has been postulated that the mechanism leading to the development of a traumatic AVF is the organized hematoma caused by damage to the vasa vasorum of the artery. Endothelial bud proliferation within the organized hematoma may create multiple endothelial-lined channels between the damaged arterial and venous systems, rather than a single connection [5, 6]. In this case, profuse bleeding occurred on the left side during pterygomaxillary disjunction; this vascular lesion was likely the source of the AVF development.

The time of detection for this condition can vary, presenting either immediately or weeks after surgery [5]. Therefore, diagnosis relies on a combination of patient-reported symptoms and confirmation through imaging studies, such as angiography [4]. The most commonly reported symptoms are a murmur or pulsatile tinnitus. Patients may also experience a pulsating sensation in the affected area, headaches, and tinnitus in one or both ears [3]. However, murmur and pulsatile tinnitus are the most common [3, 4], as in our patient's case, who reported a pulsating sensation and fremitus immediately postoperatively. These symptoms are caused by the diversion of blood from the high-pressure arterial system to the low-pressure venous system. Blood entering through the arteriovenous tract impacts the arterial endothelial walls, creating turbulent flow. This turbulence generates a resonance that intensifies or is perceived more intensely during systole [5, 6]. These symptoms have been reported to decrease or disappear with carotid compression [4]. Although this maneuver was not performed in our patient, he reported that the murmur became more noticeable with increased heart rate during physical activity. The murmur was even audible without a stethoscope.

Imaging typically reveals a contrast-enhancing mass with robust blood supply from nearby vessels. This mass will lose contrast during diastole and will not grow over time, unlike a pseudoaneurysm, which usually enlarges and shows persistent contrast enhancement even after the arterial phase of angiography [5].

The differential diagnosis includes hematomas, posttraumatic abscesses, and pseudoaneurysms, all of which can cause symptoms similar to AVFs [5].

Head and neck AVFs require either surgical intervention or endovascular embolization for proper management [9, 10]. Both procedures are aimed at closing the fistula while preserving surrounding blood flow [9]. However, endovascular embolization is generally the preferred treatment for definitive closure of traumatic or iatrogenic AVFs in the maxillofacial region due to its effectiveness for lesions of this location and size [4].

Endovascular embolization can be performed by placing stents, balloons, coils, or other sclerosing agents. Access for embolization can be obtained via direct puncture in the cervicofacial region or remotely through large vessels [9], as in our patient's case, where femoral access was used. Coils, introduced in 1975, reduce blood flow within the AVF. Currently, platinum coils are used, which allow for better control, easier placement, and the possibility of repositioning or replacement [9].

One advantage of endovascular embolization over surgery is that more distal vessels supplying the bleeding source can be obliterated while more proximal vessels remain unaffected [5, 6, 9, 10]. This is particularly relevant after orthognathic surgery to avoid further compromising an already reduced vascular supply, which could contribute to the development of avascular necrosis of the jaw [3, 5, 6, 10].

This case illustrates the importance of adequate soft tissue protection during orthognathic surgery, especially during maxillary osteotomies for pterygomaxillary suture disjunction. It also emphasizes the importance of considering surgical landmarks and understanding the anatomical structures at risk for injury during this procedure, which can lead to AVF formation. Although this complication is rare, the ever-present risk must be acknowledged. Timely diagnosis and treatment are crucial to minimizing potential complications.

## Data Availability

The data that support the findings of this study are available from the corresponding author upon reasonable request.
